# Serum albumin level as a potential marker for deciding chemotherapy or best supportive care in elderly, advanced non-small cell lung cancer patients with poor performance status

**DOI:** 10.1186/s12885-017-3814-3

**Published:** 2017-11-28

**Authors:** Satoshi Ikeda, Hiroshige Yoshioka, Satoshi Ikeo, Mitsunori Morita, Naoyuki Sone, Takashi Niwa, Akihiro Nishiyama, Toshihide Yokoyama, Akimasa Sekine, Takashi Ogura, Tadashi Ishida

**Affiliations:** 1 Department of Respiratory Medicine, Kurashiki Central Hospital, Miwa 1-1-1, Kurashiki-city, 710-8602 Japan; 2 Department of Respiratory Medicine, Kanagawa Cardiovascular and Respiratory Center, Tomioka-Higashi 6-16-1, Kanazawa-ku, Yokohama-city, 236-0051 Japan

**Keywords:** Non-small cell lung cancer, Elderly, Performance status, Albumin, Hypoalbuminemia

## Abstract

**Background:**

There have been few data on the chemotherapy in elderly advanced non-small cell lung cancer (NSCLC) patients with poor performance status (PS), and usefulness of chemotherapy for such patients remains unclear. The objective of this study was to identify factors that predicted the survival benefit of chemotherapy.

**Methods:**

All consecutive elderly patients (≥75 years) with advanced NSCLC, Eastern Cooperative Oncology Group PS ≥2, EGFR mutation wild type/unknown, and newly diagnosed from January 2009 to December 2012 at a tertiary hospital were retrospectively reviewed.

**Results:**

We enrolled 59 patients, and 31 patients received at least one chemotherapy regimen (chemotherapy group). However, 28 patients received best supportive care (BSC) alone (BSC group). The proportion of PS 2 and serum albumin levels was significantly higher in the chemotherapy group than in the BSC group. In the chemotherapy group, log-rank testing did not show statistically significant differences in overall survival (OS) between the single-agent therapy group and carboplatin-based doublet therapy group; however, the OS of patients receiving chemotherapy for only 1 cycle (early termination) was significantly shorter than patients receiving chemotherapy for ≥2 cycles. Hypoalbuminemia was not only a risk factor for the early termination of chemotherapy but also an independent prognostic factor in the chemotherapy group. A receiver operating characteristic curve analysis showed that the best cut-off value was 3.40 g/dL. In patients with serum albumin levels ≥3.40 g/dL, OS was significantly better in the chemotherapy group than in the BSC group (*p* = 0.0156), however, patients with serum albumin levels <3.40 g/dL exhibited poor prognosis regardless of the presence or absence of chemotherapy.

**Conclusion:**

In the elderly NSCLC patients with poor PS, serum albumin levels may help identify certain patient populations more likely to receive a survival benefit of systemic chemotherapy.

**Electronic supplementary material:**

The online version of this article (10.1186/s12885-017-3814-3) contains supplementary material, which is available to authorized users.

## Background

Among patients newly diagnosed with non-small cell lung cancer (NSCLC) in developed countries, approximately 50% are ≥70 years at the time of diagnosis [1], and 30%–40% are with an Eastern Cooperative Oncology Group (ECOG) performance status (PS) ≥ 2 [2]. Because older age and poor PS have often been related to the increased risk of toxicity associated with cytotoxic chemotherapy, such patients have often been excluded from clinical trials. To note, some randomized phase 3 trials of single-agent therapy have been conducted for elderly, advanced NSCLC patients. In the Elderly Lung Cancer Vinorelbine Italian Study (ELVIS), median overall survival (OS) was significantly better in the vinorelbine group than that in the best supportive care (BSC) group [3, 4]. The Multicenter Italian Lung Cancer in the Elderly Study (MILES) revealed that median OS in the gemcitabine group was almost equal to that in the vinorelbine group [5]. Subsequently, the WJTOG9904 trial [6] showed that patients treated with docetaxel had a significantly higher response rate and better progression-free survival (PFS) compared with patients taking vinorelbine. However, the difference in OS was not statistically significant, and severe neutropenia was more common with docetaxel. In addition, trials of platinum-based doublet therapy have also been conducted in elderly patients. In a French Intergroup Study (IFCT-0501), OS was significantly betterin the carboplatin plus weekly paclitaxel group than that in the single-agent therapy (gemcitabine or vinorelbine) group [7]. However, grade ≥ 3 neutropenia and treatment-related death was more common with carboplatin plus weekly paclitaxel compared with single-agent therapy. Based on these trial results, single-agent therapy (docetaxel, gemcitabine, or vinorelbine) was recommended as first-line treatment for elderly, advanced NSCLC patients without known driver mutations, and carboplatin-based doublet therapy may be a viable option in patients deemed able to tolerate such therapy. However, little is known concerning chemotherapy in elderly, advanced NSCLC patients with poor PS, and the usefulness of chemotherapy for such patients remains unclear. Moreover, elderly patients who are enrolled in clinical trials represent a carefully selected subset. In clinical practice, elderly patients are a more heterogeneous population, with baseline organ dysfunctions and variable comorbidities, and the PS alone is not sufficient enough to account for the heterogeneity within elderly patients. It is critically important to identify patient populations that can receive a survival benefit of systemic chemotherapy in elderly patients with poor PS. In the present study, we retrospectively reviewed consecutive elderly patients (≥75 years of age) with advanced NSCLC and with poor PS (ECOG PS ≥ 2) to identify factors that predict the survival benefit of cytotoxic chemotherapy.

## Methods

### Patients and settings

All consecutive patients enrolled were (1) pathologically or cytologically confirmed NSCLC; (2) at stage IIIB or IV according to the 7th edition TNM classification; (3) ≥75 years of age; (4) with an Eastern Cooperative Oncology Group (ECOG) performance status (PS) ≥ 2; (5) with an epidermal growth factor receptor mutation wild type or unknown status; and (6) newly diagnosed at the Kurashiki Central Hospital (Kurashiki city, Okayama, Japan) from January 2009 to December 2012. The exclusion criteria included clinical diagnosis of lung cancer without pathological or cytological confirmation. In patients with ECOG PS ≥ 3, chemotherapy could be carried out only when the patient was diagnosed as treatable and tolerable for chemotherapy by the attending physician, and the patient and family were strongly hoping for the chemotherapy, even though they knew all the risks. This study has been carried out in accordance with the Declaration of Helsinki. The Ethics Committee of the Kurashiki Central Hospital approved the study protocol, and patient consent was waived because this was a retrospective study and anonymity was secured.

### Clinical and laboratory findings

Clinical and laboratory data used in this study were retrieved from patient medical records and included age; gender; the ECOG PS; smoking status; comorbidities; tumor histology; cancer stage; major diameter of the primary site; metastatic organs (brain, bone, liver, and adrenal gland); laboratory data such as white blood cell, neutrophil, and lymphocyte counts as well as hemoglobin, albumin, lactate dehydrogenase, serum calcium, and C-reactive protein levels; treatment status; progression free survival (PFS) of initial treatment; and OS. The OS was defined as the length of time from the date of diagnosis to death of any cause.

### Statistical analysis

Categorical data are presented as numbers (percentages), whereas continuous data are presented as medians (interquartile ranges). Fisher’s exact test was used to compare categorical data, and the Mann–Whitney U test was used to compare continuous data. Cumulative survival probabilities were estimated using the Kaplan-Meier method. The log-rank test was used to compare survival among patient groups. A multivariate analysis using a Cox proportional hazard model was performed to identify the factors associated with survival. A multivariate logistic regression analysis was performed to verify the risk factor for a categorical dependent variable. The factors with *p*-values <0.05 in univariate analysis were selected as candidate factors of multivariate analysis. A receiver operating characteristic (ROC) curve analysis was used to determine the optimal cut-off values for the risk factor; values with maximum joint sensitivity and specificity were selected. A *p*-value of <0.05 was considered statistically significant.

## Results

### Baseline characteristics and prognoses in the study population

**Fig. 1:**
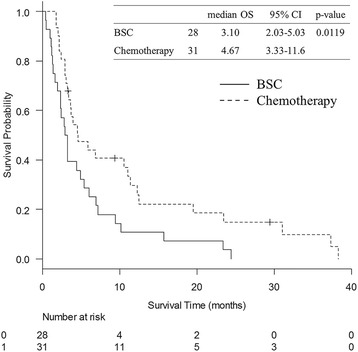
A comparison of survival curves between chemotherapy and BSC groups. A comparison of survival curves is shown. The overall survival (OS) was better in the chemotherapy group than in the BSC group

In the present study, 59 patients were enrolled. Thirty-one patients received at least one chemotherapy regimen (chemotherapy group), whereas 28 patients received best supportive care (BSC) alone (BSC group). Patients’ characteristics are summarized in Table 1. The proportion of PS 2, lymphocyte count, and serum albumin level were significantly higher in the chemotherapy group than in the BSC group. No significant differences were observed regarding other clinical and laboratory data. A comparison of survival curves is shown in Fig. 1. The OS was better in the chemotherapy group than in the BSC group (median OS of 4.7 months and 3.1 months, *p* = 0.0119).

**Table 1:** Baseline characteristics of the study population

	Chemotherapy (*N* = 31)	Best supportive care (*N* = 28)	*p*-value
Age	78.0 [76.5–80.0]	80.5 [77.0–84.3]	0.118
Gender (male/female)	4 / 27	4 / 24	1.00
ECOG Performance Status (2/3/4)	18/12/1	8/15/5	0.0350
Smoking history	30 (96.8%)	22 (78.6%)	0.0870
Brinkman Index	1100 [780–1550]	800 [420–1395]	0.113
Comorbidities			
Emphysema (%)	26 (83.9%)	20 (71.4%)	0.348
Interstitial pneumonia (%)	1 (3.2%)	1 (3.6%)	1.00
Diabetes mellitus (%)	13 (41.9%)	1 (3.6%)	0.00100
Histology (Non-Squamous/Squamous)	6 / 25	6 / 22	1.00
Staging (IIB/IV)	5 / 26	2 / 26	0.428
Major diameter of the primary site	39.5 [27.5–65.0]	52.0 [40.5–74.0]	0.0740
Metastatic organ			
Brain (%)	6 (19.4%)	4 (14.3%)	0.734
Bone (%)	10 (32.3%)	6 (21.4%)	0.393
Liver (%)	3 (9.7%)	3 (10.7%)	1.00
Adrenal gland (%)	4 (12.9%)	1 (3.6%)	0.356
Laboratory data			
White blood cell count	7800 [6450–9700]	7700 [6175–11,925]	0.802
Neutrophil count	5336 [4502–6684]	5988 [4513–8929]	0.362
Lymphocyte count	1396 [1148–1721]	1086 [848–1432]	0.0370
Hemoglobin	12.4 [11.0–14.0]	12.0 [11.5–13.2]	0.885
Albumin	3.60 [3.20–3.95]	3.30 [2.85–3.62]	0.0460
Lactate dehydrogenase	214 [188–247]	247 [206–277]	0.141
Calcium	9.20 [9.00–9.55]	9.00 [8.47–9.33]	0.093
C-reactive protein	1.81 [0.66–3.92]	3.20 [0.57–7.59]	0.391

Categorical data are presented as numbers (percentages) whereas continuous data are presented as medians (interquartile ranges). Fisher’s exact test was used to compare categorical data, and the Mann–Whitney U test was used to compare continuous data

Abbreviations: *ECOG* Eastern Cooperative Oncology Group

### Treatment details and prognosis in the chemotherapy group

**Fig. 2:**
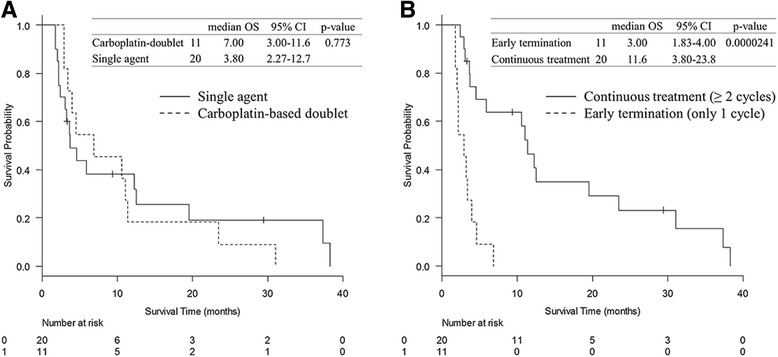
Log-rank testing in the chemotherapy group. Log-rank testing did not show statistically significant differences in median overall survival (OS) between single-agent therapy and carboplatin-based doublet therapy groups (**a**). To note, the OS of patients who received chemotherapy for only 1 cycle was significantly shorter than those of patients who received chemotherapy for ≥2 cycles (**b**)

Treatment details in the chemotherapy group are shown in Table 2. Twenty of the 31 patients (64.5%) received single-agent therapy, whereas 11 of the 31 patients (35.5%) received carboplatin-based doublet therapy. Patients who received carboplatin-based doublet therapy had higher response rates, and the median PFS values were better. No significant differences were observed in the disease control rate and median number of treatment cycles. An adverse event was the most common cause of cessation in patients receiving single-agent therapy, whereas, in patients receiving carboplatin-based doublet therapy, completion of 4–6 courses was the most common, followed by an adverse event. With regard to OS, log-rank testing did not show statistically significant differences between the single-agent therapy and carboplatin-based doublet therapy groups (median OS of 3.80 months and 7.00 months, *p* = 0.773) (Fig. 2a). On the other hand, the OS of patients receiving chemotherapy for only 1 cycle was significantly shorter than patients receiving chemotherapy for ≥2 cycles (median OS of 3.0 months and 11.6 months, *p* = 0.0000241) (Fig. 2b).

**Table 2:** Treatment details and prognoses of first-line chemotherapy

	Single-agent (*N* = 20)	Platinum doublet (*N* = 11)
Regimen		
Gemcitabine	8 (40.0%)	0
Vinorelbine	6 (30.0%)	0
Docetaxel	5 (25.0%)	0
Pemetrexed	1 (5.0%)	0
Carboplatin + weekly paclitaxel	0	9 (81.8%)
Carboplatin + gemcitabine	0	1 (9.1%)
Carboplatin + S-1	0	1 (9.1%)
Response rate (%)	0	45.4%
Disease control rate (%)	55.0%	54.5%
Progression free survival (month)	2.87 [0.60–7.27]	5.43 [1.58–8.07]
Number of treatment cycles	2.00 [1.00–2.25]	3.00 [1.00–4.00]
Early termination (only 1 cycle) (%)	7 (35.0%)	4 (36.4%)
Cause of cessation		
Adverse event	11 (55.0%)	4 (36.4%)
Deterioration of physical condition	5 (25.0%)	0
Completion of 4–6 cycles	0	5 (45.5%)
Progressive disease	3 (15.0%)	1 (9.1%)
Patient’s request	1 (5.0%)	1 (9.1%)

Categorical data are presented as numbers (percentages) whereas continuous data are presented as medians (interquartile ranges)

### Risk factors for the early termination of chemotherapy

Eleven patients received chemotherapy for only 1 cycle (early termination group), whereas 20 patients received chemotherapy for ≥2 cycles (continuous treatment group). When comparing the clinical and laboratory data between two groups (Additional file 1 Table S1), the incidence of bone metastasis was higher and serum albumin levels were lower in the early termination group than in the continuous treatment group. No significant differences were observed for any other clinical and laboratory data.

A logistic regression analysis was performed to verify the risk factor for the early termination of chemotherapy (Table 3). In univariate analysis, serum albumin level and the existence of bone metastasis, all with *p*-values <0.05, were selected as candidate risk factors. A multivariate analysis showed that low serum albumin level and the existence of bone metastasis were significantly associated with the early termination of chemotherapy (*p* = 0.0493 and 0.0174, respectively).

**Table 3:** Logistic regression analysis verifying the risk factors for early termination of chemotherapy (*N* = 31)

	Odds ratio	95% confidence interval	*p*-value
Univariate analysis			
Age	0.93	0.722–1.20	0.575
ECOG Performance status = 2	0.449	0.100–2.01	0.295
Brinkman Index	1.00	0.999–1.00	0.655
Emphysema	0.794	0.112–5.66	0.818
Diabetes melitus	2.23	0.497–10.0	0.295
Squamous cell carcinoma	0.889	0.135–5.85	0.902
Major diameter of the primary site	1.01	0.973–1.04	0.672
Brain metastasis	2.12	0.349–13.0	0.414
Bone metastasis	9.92	1.75–56.3	0.00961
Liver metastasis	0.900	0.0723–11.2	0.935
Adrenal gland metastasis	7.12	0.640–79.3	0.110
Carboplatin-based doublet therapy	1.06	0.229–4.92	0.939
Lymphocyte count	1	0.999–1.00	0.866
Hemoglobin	0.637	0.392–1.04	0.0691
Albumin	0.117	0.0168–0.811	0.0299
Lactate dehydrogenase	1.01	0.999–1.02	0.0979
Calcium	1.19	0.632–2.24	0.59
C-reactive protein	1.15	0.897–1.48	0.267
Multivariate analysis			
Bone metastasis	10.9	1.52–77.9	0.0174
Albumin	0.0886	0.00791–0.992	0.0493

In the univariate analysis, serum albumin level and the existence of bone metastasis, all with *p*-values <0.05, were selected as candidate risk factors. A multivariate analysis showed that the association between serum albumin level and the existence of bone metastasis with early termination of chemotherapy were statistically significant

Abbreviations: *ECOG* Eastern Cooperative Oncology Group

### The prognostic factors in the chemotherapy group

An analysis using a Cox proportional hazard model was performed to verify the prognostic factor associated with survival in the chemotherapy group (Table 4). In univariate analysis, serum albumin level, number of cycles, the existence of bone metastasis, and the existence of adrenal gland metastasis, all with *p*-values <0.05, were selected as candidate factors. A multivariate analysis identified the serum albumin level as an independent factor associated with survival [hazard ratio: 0.174; 95% confidence interval (CI): 0.0610–0.495; *p* = 0.00104].

**Table 4:** Analysis using a Cox proportional hazard model to verify the prognostic factor associated with survival in the chemotherapy group (*N* = 31)

	Hazard ratio	95% confidence interval	*p*-value
Univariate analysis			
Age	0.995	0.884–1.12	0.929
ECOG Performance status = 2	0.994	0.453–2.18	0.987
Brinkman Index	0.999	0.998–1.00	0.104
Emphysema	0.649	0.241–1.74	0.391
Diabetes mellitus	0.990	0.448–2.189	0.980
Squamous cell carcinoma	1.15	0.420–3.12	0.792
Major diameter of the primary site	1.02	0.997–1.03	0.102
Brain metastasis	2.82	0.986–8.04	0.0533
Bone metastasis	3.07	1.24–7.57	0.0150
Liver metastasis	1.29	0.294–5.65	0.736
Adrenal gland metastasis	4.77	1.21–18.8	0.0253
Carboplatin-based doublet therapy	1.12	0.513–2.46	0.773
Number of treatment cycles	0.665	0.483–0.915	0.0122
Lymphocyte count	1.00	0.999–1.00	0.321
Hemoglobin	0.789	0.621–1.00	0.0511
Albumin	0.180	0.0694–0.465	0.000408
Lactate dehydrogenase	1.00	0.998–1.00	0.455
Calcium	1.15	0.764–1.72	0.51
C-reactive protein	1.08	0.961–1.21	0.196
Multivariate analysis			
Bone metastasis	1.98	0.666–5.90	0.2190
Adrenal gland metastasis	2.19	0.470–10.17	0.3180
Number of treatment cycles	0.744	0.518–1.07	0.110
Albumin	0.18	0.0638–0.508	0.00121

In the univariate analysis, serum albumin level, number of cycles, the existence of bone metastasis, and the existence of adrenal gland metastasis, all with *p*-values <0.05, were selected as candidate factors. A multivariate analysis identified serum albumin level as an independent factor associated with survival

Abbreviations: *ECOG* Eastern Cooperative Oncology Group

### Best cut off value for the serum albumin level

To determine the cut-off values of serum albumin level for the “early termination of chemotherapy,” an ROC curve analysis was performed. The area under the curve for the serum albumin level was 0.752 (95% CI: 0.570–0.934) and the cut-off value for which sensitivity + specificity was maximal was 3.40 g/dL (81.8% sensitivity and 70.0% specificity).

In addition, we performed a ROC curve analysis to determine the cut-off values of serum albumin level for “death within 3 months” in the chemotherapy group, which was based on the median OS of 3.1 months in the BSC group in the present study. The area under the curve for the serum albumin level was 0.739 (95% CI: 0.531–0.947) and the cut-off value for which sensitivity + specificity was maximal was also 3.40 g/dL (87.5% sensitivity and 65.2% specificity).

### Comparison of survival curves based on serum albumin levels

**Fig. 3:**
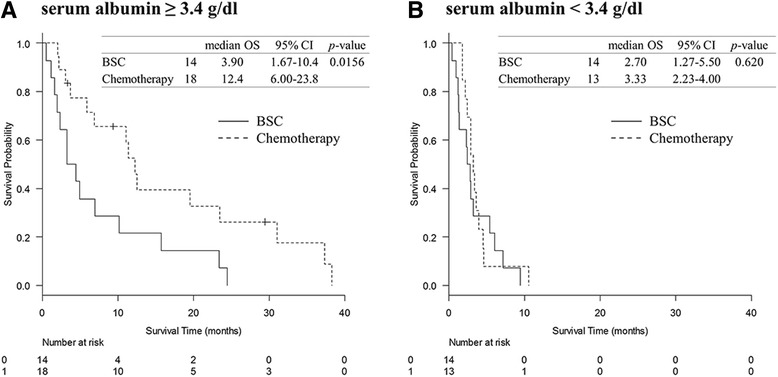
Comparison of survival curves based on serum albumin levels**.** In the patients with serum albumin levels ≥3.40 g/dL, overall survival (OS) was significantly better in the chemotherapy group than that in the BSC group (**a**); in patients with serum albumin levels <3.40 g/dL, the OS did not differ between chemotherapy and BSC groups (**b**)

We compared the survival curves between the BSC and chemotherapy groups based on the serum albumin level. For patients with serum albumin levels ≥3.40 g/dL, OS was significantly better in the chemotherapy group than that in the BSC group (respective median OS of 12.7 months and 3.9 months, *p* = 0.0156) (Fig. 3a). In patients with serum albumin levels <3.40 g/dL, the OS did not differ between the chemotherapy and BSC groups (respective median OS of 3.3 months and 2. 7 months, *p* = 0.620) (Fig. 3b).

## Discussion

The present study demonstrated the following three important clinical observations. First, the OS of the chemotherapy group was better than that of the BSC group in elderly patients with poor PS. Second, the number of treatment cycles had a larger impact on the survival benefit of chemotherapy than the decision/selection of either single-agent therapy or carboplatin-doublet therapy. Third, hypoalbuminemia was not only the risk factor for early termination of chemotherapy, but also the independent prognostic factor in the chemotherapy group.

The clinician-estimated PS is the most common method to evaluate physiologic reserve and functional status in NSCLC patients, and it is used to assess a patient’s tolerability against chemotherapy. In previous clinical trials conducted for elderly, advanced NSCLC patients, such as the ELVIS and IFCT-0501 trials [3, 4, 7], 20–30% of patients had a PS of 2, whereas almost no data were available for patients with PS ≥ 3. Given this, there is a general consensus that elderly patients with PS 2 who wish to receive treatment should be offered chemotherapy, and elderly patients with PS ≥ 3 should receive supportive care aimed at maintaining quality of life [8]. In the present study, because of the differences in the baseline characteristics between the chemotherapy and BSC groups, it cannot be simply considered that chemotherapy prolonged OS in elderly patients with poor PS. However, meta-analysis of the clinical trials comparing chemotherapy and BSC for advanced NSCLC demonstrated that chemotherapy improves OS even in patients with poor PS [9]. Moreover, when comparing patients with PS 2 and PS ≥ 3 in the chemotherapy group of the present study, there were no significant differences in the median number of initial treatment cycles (2 cycles each), disease control rates of the initial treatment (64.7% in PS 2 and 66.7% in PS ≥ 3), and median OS (6.50 months in PS 2 and 4.00 months in PS ≥ 3, *p* = 0.987), regardless of the chemotherapy regimen. These results indicated that PS tends to be insufficient for assessing tolerability against chemotherapy and prognosis in elderly patients. Thus, there would be a certain population within elderly patients with poor PS to benefit via survival due to systemic chemotherapy. Especially in elderly patients, PS easily fluctuates based on various factors, such as pain caused by cancer; thus, treatment decision-making should not be made based on temporal PS alone.

When performing chemotherapy, the optimal regimen for elderly patients with poor PS remains controversial. Carboplatin-based doublet therapy is clearly superior to single-agent therapy regarding antitumor effect, but it results in higher toxicity. In the present study, the response rate was higher and PFS was better in the carboplatin-doublet patient group than the response rate and PFS in the single-agent group (Table 2). However, there were no significant differences observed in the OS between the two groups (Fig. 2a). In previous randomized control trials designed for elderly populations tasked to compare non-platinum single agent and platinum-doublet therapies, only the IFCT-0501 trial showed the survival benefit of carboplatin plus weekly paclitaxel, even in patients with PS 2 [7], whereas other trials did not show statistically significant differences in OS [10–12]. In a real-world setting, patients were more heterogeneous and the proportion of frail patients was higher than those in clinical trials, thus the results of IFCT-0501 cannot apply entirely to the elderly population, especially patients with poor PS. The present study also revealed that the OS was significantly shorter in the early termination group than that in the continuous treatment group. Thus, for elderly patients with poor PS, consideration should be given to reasonably choose single-agent therapy, with low toxicity and continuation of as many cycles as possible.

For the treatment decision-making in elderly patients, geriatric assessment, including physical function, comorbidities, psychological state, social support, cognitive function, nutrition, and polypharmacy, is needed in conjunction with PS. Comprehensive geriatric assessment (CGA) has been adopted to evaluate elderly patients with cancer and may help identify patients who are fit and more likely to benefit from chemotherapy [13]. However, the recent ESOGIA-GFPC-GECP 08–02 trial in elderly patients with advanced NSCLC failed to show a survival benefit of CGA-based strategy in spite of significantly fewer treatment failures attributed to toxicity [14]. In the present study, hypoalbuminemia was significantly associated with early termination of chemotherapy, and the patients without hypoalbuminemia received a significant survival benefit from chemotherapy. As one of the factors contributing to early termination, hypoalbuminemia was reported to correlate with grade ≥ 3 non-hematological toxicity in elderly NSCLC patients [15]. On the other hand, the present study revealed that hypoalbuminemia was independently associated with survival in the chemotherapy group, and patients with hypoalbuminemia exhibited poor prognosis regardless of presence or absence of chemotherapy. Previous epidemiological works dissecting the association between pretreatment serum albumin levels and survival in NSCLC revealed that higher serum albumin levels were associated with better survival [16–23]. From these results, it was speculated that serum albumin level predicts the survival benefit of chemotherapy in elderly, advanced NSCLC patients with poor PS. In the CGA measurement tools, body mass index was often used for the assessment of nutrition status, whereas the serum albumin level was rarely used. The assessment tool including the serum albumin level, such as the Chemotherapy Risk Assessment Scale for High age (CRASH) score [15], may help identify patients more likely to benefit from chemotherapy.

A limitation of the present study was the retrospective single-center study design. Additionally, the number of included patients was small and the distribution of patients may have been skewed. There is a need to accumulate more cases from a plurality of hospitals and conduct further investigations for the validation of the present results. Factors associated with geriatric assessment, such as psychological state, social support, and cognitive function, were not fully evaluated. We might have to consider that prolongation of OS as an optimal endpoint for elderly, advanced NSCLC patients with poor PS.

## Conclusions

In elderly, advanced NSCLC patients with poor PS, serum albumin levels may help identify certain populations more likely to receive a survival benefit of systemic chemotherapy.

## Additional file

Additional file 1: Table S1. Comparison of clinical and laboratory data between the early termination group and the continuous treatment group (DOCX 14 kb)

## Abbreviations

BSC: best supportive care
CGA: Comprehensive geriatric assessment
CRASH: Chemotherapy risk assessment scale for high age
ECOG: Eastern Cooperative Oncology Group
ELVIS: Elderly lung cancer vinorelbine italian study
MILES: Multicenter Italian Lung Cancer in the Elderly Study
NSCLC: non-small cell lung cancer
OS: overall survival
PFS: progression-free survival
PS: performance status
ROC: receiver operating characteristic
