# Supplementary material for: Serum albumin level as a potential marker for deciding chemotherapy or best supportive care in elderly, advanced non-small cell lung cancer patients with poor performance status
Source: BMC Cancer. 2017 Nov 28;17:797. doi: 10.1186/s12885-017-3814-3 (PMC5704395; doi:10.1186/s12885-017-3814-3)
Supplement: Additional file 1: Table S1. — Comparison of clinical and laboratory data between the early termination group and the continuous treatment group (DOCX 14 kb) [file 12885_2017_3814_MOESM1_ESM.docx]

**Supplemental Table 1. Comparison of clinical and laboratory data between the early termination group and the continuous treatment group.**

|  | Early termination (N = 11) | Continuous treatment (N = 20) | *p*-value |
| --- | --- | --- | --- |
| Age | 78.0 [76.0–79.5] | 78.5 [76.8–80.3] | 0.493 |
| Gender (male/female) | 11/0 | 4/16 | 0.269 |
| ECOG Performance Status (2/3/4) | 5/6/0 | 13/6/1 | 0.449 |
| Smoking history | 11 (100%) | 19 (95.0%) | 1 |
| Brinkman Index | 1100 [775–1480] | 1120 [833–1625] | 0.741 |
| Comorbidities |  |  |  |
| Emphysema (%) | 9 (81.8%) | 17 (85.0%) | 1 |
| Interstitial pneumonia (%) | 1 (9.1%) | 0 | 0.355 |
| Diabetes mellitus (%) | 6 (54.5%) | 7 (35.0%) | 0.449 |
| Histology (Non-Squamous/Squamous) | 9/2 | 16/4 | 1 |
| Staging (IIIB/IV) | 0/11 | 5/15 | 0.133 |
| Major diameter of the primary site | 41.0 [33.5–57.5] | 37.0 [25.0–72.0] | 0.335 |
| Metastatic organ |  |  |  |
| Brain (%) | 3 (27.3%) | 3 (15.0%) | 0.638 |
| Bone (%) | 7 (63.6%) | 3 (15.0%) | 0.013 |
| Liver (%) | 1 (9.1%) | 2 (10.0%) | 1 |
| Adrenal gland (%) | 3 (27.3%) | 1 (5.0%) | 0.115 |
| Laboratory data |  |  |  |
| White blood cell count | 8700 [7500–11550] | 7500 [6400–9025] | 0.186 |
| Neutrophil count | 6586 [5100–8668] | 4872 [4343–6016] | 0.148 |
| Lymphocyte count | 1183 [1028–2056] | 1415 [1265–1666] | 0.591 |
| Hemoglobin | 11.3 [10.6–12.5] | 13.0 [11.6–14.3] | 0.066 |
| Albumin | 3.30 [3.10–3.35] | 3.75 [3.38–4.05] | 0.022 |
| Lactate dehydrogenase | 247 [200–351] | 199 [184–234] | 0.121 |
| Calcium | 9.00 [8.85–9.30] | 9.30 [9.00–9.67] | 0.245 |
| C-reactive protein | 3.41 [1.83–4.49] | 1.22 [0.48–2.92] | 0.069 |

Abbreviations: ECOG = Eastern Cooperative Oncology Group.
